# NO- and S-nitrosothiols-dependent relaxation in large and small resistance blood vessels

**DOI:** 10.3389/fphys.2026.1801670

**Published:** 2026-04-13

**Authors:** Mauricio A. Lillo, Annie Beuve

**Affiliations:** Department of Pharmacology, Physiology and Neuroscience, Rutgers-New Jersey Medical School, Newark, NJ, United States

**Keywords:** calcium signaling, EDH, nitric oxide, resistance arteries, S-nitrosylation and S-nitrosation, soluble guanylyl cyclase (GC1)

## Abstract

Nitric oxide (NO) has long been viewed as the principal endothelium-derived vasodilator through activation of soluble guanylyl cyclase (GC1) and cGMP-dependent signaling in vascular smooth muscle. However, accumulating evidence indicates that this canonical NO-GC1-cGMP pathway predominates in large conduit arteries, whereas regulation of vascular tone and blood pressure in the microcirculation relies largely on endothelium-derived hyperpolarization (EDH) with NO acting primarily as a redox signal rather than a freely diffusing gas. Here, we review emerging mechanisms that integrate NO-derived nitrosative signaling with EDH, highlighting the central role of protein S-nitrosation and transnitrosation in shaping endothelial excitability. A central focus of this review is the newly-identified endothelial role of GC1 beyond its canonical smooth muscle function. We summarize recent findings demonstrating that GC1 acts as a redox hub through S-nitrosation of a critical cysteine residue (cys), enabling selective transnitrosation of downstream targets that regulates endothelial Ca^2+^ influx, KCa channel activation, and EDH-dependent vasodilation independently of cGMP production. Finally, we examine how extravascular components-including perivascular adipose tissue and red blood cells-modulate EDH signaling under physiological and pathological conditions.

## Introduction

There has been some confusion regarding the role(s) of NO (the free radical ^•^NO) and its reactive derivatives, including the nitrosating species in the vasorelaxation of blood vessels. ^•^NO is a gas that diffuses freely across membranes and binds to the heme of proteins. The main hemoprotein receptor, which is also an enzyme, is the soluble guanylyl cyclase (GC1) (Denninger and Marletta, 1999). NO has to bind to the heme of GC1 to stimulate the catalytic activity of GC1, which is to convert Mg^2+^-GTP into cGMP. In conduit arteries, this is the main mechanism by which vasorelaxation is achieved. NO also binds to hemoglobin/myoglobin but with a lower rate than NO binding to GC1 (Wu et al., 2022). The binding of NO to other hemoproteins such as GAPDH is also critical for cellular functions, including transfer of the heme-NO of GAPDH to apo-GC1 (Dai and Stuehr, 2025). Recently it was proposed that a more stable heme-nitrosyl complex (NO-ferroheme) could support vasorelaxation by binding to an apo-GC1 form (DeMartino et al., 2023; Kleschyov et al., 2023). NO as a gas cannot directly react with a thiol and needs to be modified first by an oxidant or radical to react with cysteine-thiols to form S-nitrosothiol (SNO) groups, or with another molecule of NO to form the S-nitrosating agent N_2_O_3_. The addition of this NO reactive moiety to the cysteines (cys) of proteins is called S-nitrosation or S-nitrosylation (Hess et al., 2005; Broniowska and Hogg, 2012). In resistance arteries, vasodilation is governed by endothelial Ca^2+^ microdomains, electrical coupling through myoendothelial junctions, and hyperpolarization mediated by SKCa and IKCa channels, with NO acting primarily as a “NO-derived oxidant” rather than a freely diffusing gas. Key EDH effectors-including connexin 43 gap junctions and hemichannels, TRPV4 Ca^2+^ channels, and other Ca^2+^-handling proteins are regulated by targeted S-nitrosation events within specialized endothelial microdomains. Herein, we will distinguish these two forms of NO signaling and their biological functions with an emphasis on GC1’ ability to transfer nitrosothiols to other specific cys of other proteins in a reaction called transnitrosation.

## Different endothelium-dependent relaxations in blood vessels

**Figure 1:**
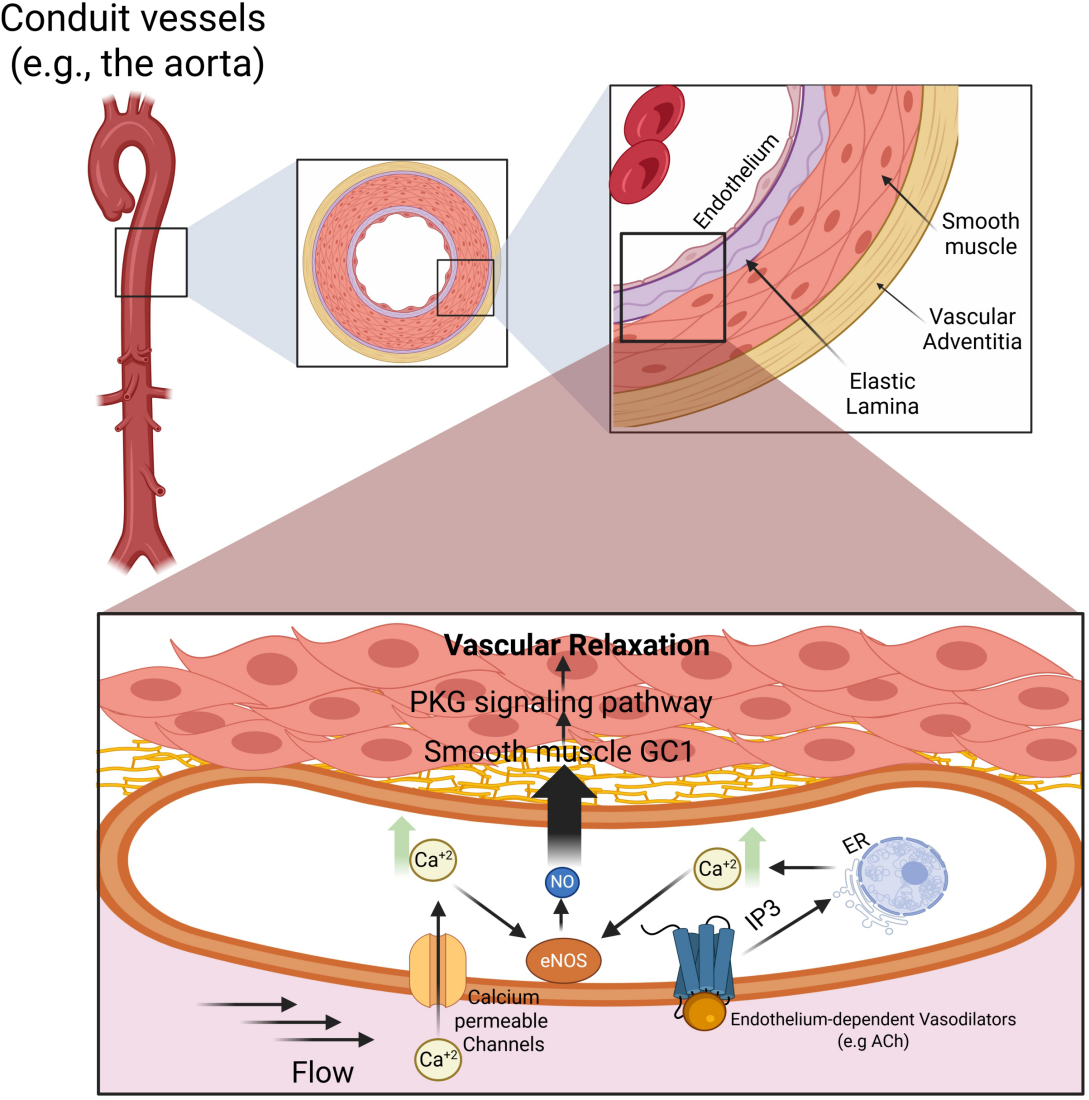
Canonical NO–GC1–cGMP signaling mediates vascular relaxation in conduit arteries. Schematic representation of signaling mechanisms underlying vascular relaxation in large conduit vessels, exemplified by the aorta. Conduit arteries are characterized by a thick smooth muscle layer, a prominent elastic lamina, and limited myoendothelial coupling. Endothelial stimulation by flow or agonists such as acetylcholine induces Ca^2+^ influx and intracellular Ca^2+^ release, activating endothelial nitric oxide synthase and promoting NO production. As a freely diffusible gas, NO readily crosses cellular membranes and reaches the underlying smooth muscle cells, where it binds to soluble guanylyl cyclase, stimulating cyclic guanosine monophosphate generation and protein kinase G activation. This signaling cascade reduces smooth muscle intracellular Ca^2+^ levels and Ca^2+^ sensitivity, leading to smooth muscle relaxation and vascular relaxation. In conduit arteries, this NO–GC1–cGMP–PKG pathway represents the dominant mechanism governing vascular relaxation and arterial compliance.

**Figure 2:**
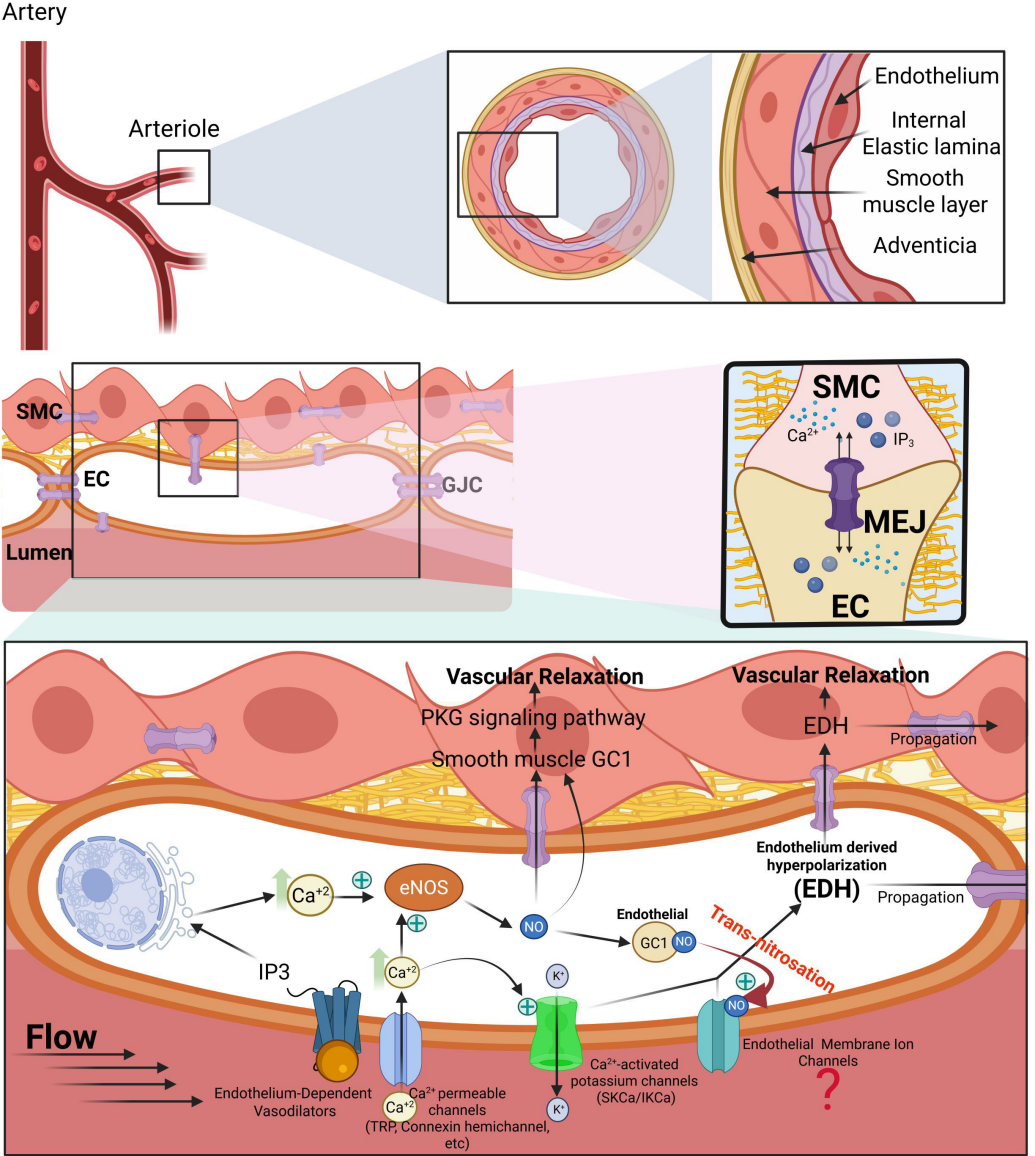
Distinct mechanisms of vascular relaxation in resistance arteries: integration of NO signaling and EDH. Schematic representation of the structural and signaling features that define vascular relaxation in resistance arteries. Upper panel: Arterioles are characterized by a thin smooth muscle layer, a prominent internal elastic lamina perforated by myoendothelial junctions (MEJs), and close electrical and biochemical coupling between endothelial cells (ECs) and smooth muscle cells (SMCs). Endothelial stimulation by flow or agonists induces Ca^2+^ influx and intracellular Ca^2+^ release, activating eNOS and generating NO. In resistance arteries, NO contributes minimally as a freely diffusible vasodilator and instead acts predominantly as a redox signal within the endothelium. Lower panel: Localized Ca^2+^ microdomains generated by Ca^2+^-permeable channels, including TRP channels and connexin hemichannels, selectively activate SKCa and IKCa channels, leading to endothelial hyperpolarization. This hyperpolarizing current spreads longitudinally through endothelial gap junctional coupling and radially to the underlying smooth muscle through MEJs, resulting in smooth muscle hyperpolarization, closure of voltage-operated Ca^2+^ channels, and vascular relaxation. In parallel, NO-dependent S-nitrosation and transnitrosation events modulate key endothelial ion channels and signaling proteins, further stabilizing EDH signaling. The figure highlights the dual role of GC1 in resistance arteries. While smooth muscle GC1 supports a minor cGMP-dependent component of relaxation, endothelial GC1 is proposed to function as a redox regulator that orchestrates localized nitrosothiol signaling, linking Ca^2+^ microdomains to endothelial electrical activity. Together, these mechanisms position EDH as the dominant pathway governing vascular relaxation in the microcirculation.

The NO signaling leading to relaxation in smooth muscle cells is function of the type of arteries where it takes place (Wenceslau et al., 2021). In the conduit arteries, NO acts via stimulation of the soluble guanylyl cyclase’ catalytic production of cGMP, which in turn induces relaxation by activating protein kinase G (PKG). In the smaller arteries (resistance arteries, arterioles, diameter <250μm), NO acquires reactivity and acts via induction of hyperpolarization due to activation of K^+^ channels, whereas the NO-cGMP pathway has little or no involvement. One reason is linked to the difference in structure between conduit arteries and resistance arteries (**Figures 1** and **2**, respectively). In addition to multiple SMC layers in the conduit arteries compared to one or two layers in arterioles, the endothelial cells of the arterioles send projections through holes of an internal elastic lamina to communicate with the SMC via these myoendothelial junctions (MEJ). These MEJ are the main path for electrical signals between the ECs and SMC (Billaud et al., 2014). In addition, the wall composition is different between conduit arteries and resistance arteries. The conduit arteries have elastin as the major component of the extracellular matrix, hence their elasticity (**Figure 1**), while resistance arteries contain high amount of collagen conferring rigidity (**Figure 2**). These differences are reflected functionally as the conduit arteries will expand and relax with each cardiac cycle carrying blood flow into the smaller arteries with little impact on vascular resistance. In contrast, these small arteries control the blood flow, exhibiting pressure-induced constriction (myogenic tone), thus modulating peripheral resistance (**Figure 2**). However, there is still no definitive consensus regarding the mechanisms underlying regulation of blood pressure and this is due in part to the vastly different hypertensive phenotypes as a function of sex and strain in genetically modified mouse models (Scotland et al., 2005; Buys et al., 2008; Sips and Buys, 2013).

- In large conduit arteries, PKG mediates SMC relaxation by the NO-GC1-cGMP pathway (Surks et al., 1999; Lincoln et al., 2001). eNOS activation in the endothelium produces NO, which stimulates in SMC the production of cGMP by binding to GC1 with nanomolar affinity (Bellamy et al., 2002). cGMP-dependent PKG activation, in turn, induces vasorelaxation of SMC by reducing intracellular Ca^2+^ and decreasing myofilaments Ca^2+^ sensitivity (Hofmann et al., 2000). It was shown that in a mouse model lacking PKG, mean arterial pressure remains the same in WT and PKG knock-out mice and that acetylcholine (ACh)-induced relaxation of the mesenteric arteries was not affected in this KO mouse model (Koeppen et al., 2004) (although an earlier study observed a change in systemic blood pressure in the same KO mouse model (Pfeifer et al., 1998)). This indicated that the NO-cGMP-PKG pathway, predominant in the conduit arteries, is minimally involved in the regulation of blood pressure while the small vessels vasorelaxation is mostly independent from the NO-cGMP pathway. This was confirmed in a report showing that ACh-dependent relaxation of mesenteric arteries was independent of GC1 activation (Pankey et al., 2014).

-In small resistance arteries, impairment of vasorelaxation is independent of NO, the gas, but linked to uncoupled NO synthase (Takaki et al., 2008). The reason for small resistance vasorelaxation to be independent of the NO gas production is probably linked to the overexpression of α-globin in the small arteries compared to the larger arteries. α-globin at the MEJ is efficient in scavenging NO produced by eNOS, hence reducing the bioavailability of NO in the small arteries (Straub et al., 2012). Likewise the cellular locations of eNOS and its association with caveolin in the conduit and small arteries are different and affect the activation of eNOS (Shu et al., 2015).

The microcirculation relies more heavily on electrical communication, Ca^2+^ microdomains, and hyperpolarization-based signaling collectively known as endothelium-derived hyperpolarization (EDH) (Garland et al., 2011; Garland and Dora, 2017). In this context, NO as reactive species serves mainly as a redox signal: through S-nitrosation and transnitrosation of key endoplasmic-reticulum Ca^2+^-handling proteins and plasma-membrane channels such as TRP channels and connexin (Lillo et al., 2018; Burboa et al., 2025a). The relaxation in the small arteries takes place predominantly via membrane hyperpolarization through activation of K^+^ channels, which closes voltage-operated Ca^2+^ channels VOCCs and suppresses Ca^2+^ influx. EDH is fundamentally an electrical process: endothelial Ca^2+^ increases activate SKCa and IKCa channels, generating local hyperpolarization that spreads longitudinally through the endothelial network and radially to the underlying smooth muscle through MEJ (Sonkusare et al., 2012; Penna and Pagliaro, 2025) (Hill et al., 2002; Lillo et al., 2018; Burboa et al., 2025a) (**Figure 2**).

## Role of connexins and TRPV4 in microcirculation

Several connexins (Cx37, Cx40, and Cx43) are expressed in resistance arteries, yet structural and functional evidence identifies Cx43-containing gap junctions at MEJs as the dominant pathway for electrical coupling (Sandow et al., 2003; Simon and McWhorter, 2003; Wölfle et al., 2007; Figueroa and Duling, 2009). Through Cx43, MEJs establish the endothelial resting membrane potential, facilitate the spread of hyperpolarizing current, and support the intercellular diffusion of Ca^2+^ and IP_3_ within the endothelial network and then passes into smooth muscle, suppressing voltage-dependent Ca^2+^ entry (Isakson et al., 2007; Behringer, 2017; Garland and Dora, 2017; Sedovy et al., 2023; Burboa et al., 2025b; Madeddu et al., 2025).

It is important to note that EDH signaling relies not on global elevations of endothelial Ca^2+^ but on highly localized Ca^2+^ microdomains allowing a precise and restricted activation of SKCa and IKCa channels to initiate hyperpolarization. One of the best-characterized microdomains involved in this mechanism are TRPV4 Ca^2+^ sparklets, which represent nanoscopic extracellular Ca^2+^ entry signals directly adjacent to KCa channels and other EDH effectors. In addition, Cx43 in resistance arteries forms undocked hemichannels that can generate or amplify localized Ca^2+^ signals. Recent evidence from our lab demonstrates that Cx43 hemichannel activity is tightly integrated with TRPV4 function where local Ca^2+^ elevations and NO promote S-nitrosation-dependent hemichannel opening. We showed that inhibition of Cx43 hemichannels or disruption of TRPV4-Cx43 proximity markedly blunts endothelial hyperpolarization and impairs vasodilation *in vivo* (Burboa et al., 2025a); however, TRPV4 knockout mice are not hypertensive (Filosa et al., 2013) and their resistance vessels retain substantial EDH capacity indicating that other pathways exist to support EDH (Sonkusare et al., 2012; Burboa et al., 2025a). Thus, diverse Ca^2+^ signals-TRPV4 sparklets, Piezo1 nanodomains, Cx43 hemichannel-mediated Ca^2+^ fluxes, and junctional IP_3_R events- is essential for EDH (Bauersachs et al., 1997; Vanhoutte et al., 2017; Aramide Modupe Dosunmu-Ogunbi et al., 2021).

## S-nitrosation regulation in microcirculation

For many years, NO-dependent vasodilation and EDH-mediated vasodilation were viewed as distinct, even competing, pathways in the regulation of microvascular tone. Emerging evidence now indicates that NO participates directly in EDH through protein S-nitrosation (Burboa et al., 2025a; Younis et al., 2025). Key proteins for EDH were identified as targets of S-nitrosation, modifying their activity, interaction with other proteins or/and localization. These S-nitrosated proteins include Cx43 GJ, for which S-nitrosation is essential for their opening hence their function (Lillo et al., 2019; Burboa et al., 2022; Burboa et al., 2025a; Straub et al., 2011) as well as Cx43 hemichannels, eNOS, TRPV4 channels, and potentially IP_3_ receptors and KCa channels (Shimokawa and Godo, 2020; Burboa et al., 2025a). At the MEJ, an entire system that includes GSNO reductase, eNOS and α-globin which redox state is controlled by cytochrome B5 reductase 3 (CYB5R3) (Straub et al., 2012; Straub et al., 2014) is devoted to modulation of S-nitrosation of Cx43 GJ to support EDH. Importantly, S-nitrosation of Cx43 HC is also essential for activation and interaction with TRPV4 leading to endothelial hyperpolarization. We recently showed that inhibition of Cx43 HC or disruption of TRPV4-Cx43 proximity markedly blunts EDH and impairs vasodilation *in vivo* (Burboa et al., 2025a). Our most recent research suggests that these nitrosative modifications that governs EDH requires a dedicated endothelial machinery, which could very well be modulated by soluble guanylyl cyclase (GC1) that orchestrates these targeted transnitrosation events and their location.

## Soluble guanylyl cyclase: a redox gatekeeper of endothelial hyperpolarization

For decades, the role of GC1 in vascular biology was understood almost exclusively through its canonical function in vascular smooth muscle, where NO binding to the heme of the α1/β1 heterodimer stimulates cGMP production, in turn inducing PKG-dependent relaxation. Because this mechanism is robust in conduit arteries and readily detectable in isolated smooth muscle preparations, it was assumed that GC1-mediated vasorelaxation was a smooth‐muscle-centric event, with little contribution from endothelial GC1. As a result, the potential involvement of GC1 in endothelial electrical signaling, Ca^2+^ handling, or EDH received little attention (Younis et al., 2025).

We recently demonstrated that GC1 is S-nitrosated at Cysteine 610 (C610) of the GC1 α subunit. The S-nitrosothiol group of S-nitrosated C610 (SNO-C610) can be transferred to specific Cys of other proteins, transfer that is facilitated by oxidative stress conditions (Cui et al., 2022). In particular, the oxidized form of Thioredoxin 1 (Trx1) but not the reduced form is the recipient of the SNO transfer from GC1 C610 (Cui et al., 2023). Trx1 itself is a key regulator of cellular redox and controls S-nitrosation levels of multiple proteins (Benhar et al., 2009; Sengupta and Holmgren, 2013). We showed that disrupting GC1 transnitrosation activity by mutating C610 to a serine (C610S) in the GC1 α subunit drastically reduces S-nitrosation of several proteins including Trx1 *in vitro*. Importantly, this single point mutation did not affect the NO-stimulated GC1 canonical activity, i.e., production of cGMP. Strikingly, the C610S knock-in mouse model with blunted transnitrosation activity exhibits profound endothelial dysfunction, including marked reductions in endothelial Ca^2+^ influx, blunted activation of SKCa/IKCa channels, impaired EDH, and loss of ACh-induced relaxation both ex vivo and *in vivo*, while the vasorelaxation in response to NO was unchanged (Younis et al., 2025). This clearly points to the critical *endothelial* function of GC1 in EDH and confirmed that the NO-dependent cGMP production in smooth muscle is minimally involved in vasorelaxation of resistance arteries.

The reduction of endothelial Ca^2+^ influx and blunted activation of SKCa/IKCa channels was observed in the ECs isolated from the mesenteric arteries of the KI mice. Mechanistically, this implies that the transnitrosation activity of GC1 occurs in the endothelium of the mesenteric arteries, supporting the idea that GC1 is expressed in the ECs. In fact, we showed that Trx1 S-nitrosation was significantly reduced in the primary ECs isolated from the mesenteric arteries of the KI C610S mouse model compared to WT (Younis et al., 2025). There has been few reports of GC1 expression in ECs. In bovine aorta endothelial cells, GC1 forms a complex with eNOS and HSP90 and displays the canonical NO-stimulated cGMP-production (Venema et al., 2003). Of potentially greater interest was the observation that GC1 was expressed in rat lungs ECs and that the α subunit translocated to the membrane/caveolin region after vascular endothelial growth factor (VEGF) treatment and in a calcium-dependent manner (Zabel et al., 2002), hence potentially linking Ca^2+^ signaling and GC1 function in ECs. However, these two reports only assessed the NO-cGMP canonical pathway. On the other hand, we previously observed that GC1 was S-nitrosated in human umbilical vein endothelial cells upon VEGF stimulation (Sayed et al., 2007). Thus, our next challenge will be to identify the S-nitrosated targets of GC1 in the endothelium of resistance arteries that are involved in EDH (**Figure 2**).

## Role of redox and extracellular components in EDH

NO modulates endothelial function through redox-dependent mechanisms that influence Ca^2+^ signaling and propagation of electrical signals. In resistance arteries, these nitrosative interactions in the endothelium lead to activation of KCa channels and generate EDH (Garland et al., 2011; Félétou, 2016; Garland and Dora, 2017). In this context, GC1 can be described as a regulator of endothelium function through a nitrosothiol-mediated signaling that governs EDH. In fact, its association with Trx1 could amplify this redox control but also increased the sensitivity to even small oxidative changes. At the molecular level, the interaction between GC1 and Trx1 likely establishes a localized redox microdomain that facilitates selective nitrosothiol signaling and transnitrosylation events. This coupling may enhance the efficiency and spatial precision of endothelial Ca^2+^ signaling, KCa channel activation, and electrical conduction underlying EDH. However, embedding GC1 within a Trx1-dependent redox network may also render endothelial signaling exquisitely sensitive to subtle perturbations in redox balance, such that modest oxidative stress or altered thioredoxin cycling could disproportionately disrupt nitrosative signaling and endothelial excitability.

More recently, it was proposed that the local external vascular microenvironment is important in the maintenance of this redox balance. Two major modulators are perivascular adipocyte tissue (PVAT) and red blood cells (RBCs).

-PVAT. Emerging evidence indicates (Luse et al., 2024) that communication between perivascular adipose tissue (PVAT) and the vascular wall is bidirectional. While endothelial-derived signals can shape PVAT phenotype, adipocytes surrounding resistance arteries also release paracrine factors capable of modulating endothelial function and vasomotor tone. PVAT secretes adipokines, lipids, reactive oxygen species (ROS), reactive nitrogen species (RNS) (Gao et al., 2007; Man et al., 2020), and extracellular vesicles that can influence endothelial redox balance and signaling pathways. Under physiological conditions, PVAT-derived factors may support endothelial homeostasis, including preservation of NO bioavailability and facilitation of hyperpolarization-dependent vasodilation. Redox-active mediators originating from adipocytes could, in principle, modulate the nitrosative status of key endothelial proteins such as soluble guanylyl cyclase (GC1), connexins, Ca^2+^-handling proteins, and SKCa/IKCa channels. In contrast, in metabolic disorders such as obesity and insulin resistance, PVAT becomes inflamed and hypoxic, shifting toward a pro-oxidative and pro-nitrosative phenotype. This transition promotes oxidative stress, disrupts endothelial coupling, impairs Ca^2+^-dependent activation of SKCa and IKCa channels, and attenuates EDH-mediated vasodilation (Gil-Ortega et al., 2014; Thakore and Earley, 2019).

Hydrogen peroxide (H_2_O_2_) and Hydrogen sulfide (H_2_S): Within the broader EDH framework—defined as endothelium-dependent hyperpolarization independent of NO and prostacyclin signaling—the term EDHF was originally introduced to describe a diffusible factor released from the endothelium that acts directly on vascular smooth muscle to induce hyperpolarization and relaxation (Taylor and Weston, 1988; Feletou and Vanhoutte, 2006; Feletou and Vanhoutte, 2007). While this classical model emphasized a transferable chemical mediator, the concept of EDHF has since evolved to incorporate both diffusible factors and electrical signaling mechanisms. In many vascular beds, endothelial hyperpolarization can be transmitted directly to smooth muscle via myoendothelial gap junctions, independent of a discrete chemical factor. Redox-active molecules such as H_2_O_2_ and H_2_S exemplify this conceptual expansion: although initially proposed as EDHF candidates (Zhao et al., 2001; Miura et al., 2003; Shimokawa and Matoba, 2004; Tang et al., 2005; Mustafa et al., 2009) acting on smooth muscle K^+^ channels, they may also modulate endothelial ion channel activity and intercellular coupling. These observations blur the distinction between classical EDHF signaling and EDH-mediated electrical conduction, supporting a more integrated view in which endothelial excitability is governed by a dynamic redox network. Among these, redox-sensitive molecules such as H_2_O_2_ and H_2_S have been implicated in specific vascular beds, particularly in human coronary microcirculation and resistance arteries (Miura et al., 2003; Shimokawa and Matoba, 2004; Tang et al., 2005; Mustafa et al., 2009). H_2_O_2_ has been proposed as an EDHF in coronary microvessels, where it modulates endothelial signaling, including SKCa/IKCa channel activity, and promotes smooth muscle hyperpolarization (Matoba et al., 2000; Miura et al., 2003). In contrast, H_2_S acts as a gasotransmitter that regulates vascular tone through K^+^ channel activation and redox-dependent post-translational modifications, including persulfidation (Zhao et al., 2001; Tang et al., 2005), which influence vascular reactivity. Together, these observations support the concept that endothelial hyperpolarization emerges from a dynamic redox-electrical signaling network rather than from a single diffusible factor.

-RBCs: They are now understood to play an active role in the regulation of vascular tone through ATP release by Pannexin-1 channels and redox-mediated signaling (Locovei et al., 2006; Lohman et al., 2012). During hypoxia (e.g., exercise or increased metabolic demand), red blood cells release ATP, which activates endothelial purinergic receptors and induces localized Ca^2+^ elevations. Although a direct causal link between RBC-derived ATP and EDH has not been formally established, these Ca^2+^ signals are well known to activate SKCa and IKCa channels and promote endothelial hyperpolarization. Together, these observations support the hypothesis that erythrocytes may act as active participants in the regulation of vasomotor tone by engaging endothelial Ca^2+^-dependent signaling pathways, extending their role beyond passive oxygen transport and linking local metabolic demand to microvascular reactivity (Behringer and Hakim, 2019; Kirby et al., 2021; Katona et al., 2023).

Beyond ATP signaling, red blood cells function as a redox modulator capable of transferring nitrosative bioactivity. Through the stabilization of S-nitrosothiols such as SNO-hemoglobin, RBCs do not release free NO but instead deliver S-nitrosothiol-derived NO bioactivity in an oxygen-dependent manner (Diesen et al., 2008; Premont et al., 2020) It is worth noting that this mechanism is still highly debated (Isbell et al., 2008; Katona et al., 2023). Pathophysiological states, including diabetes, hypertension, dyslipidemia, and chronic oxidative stress reduce ATP release by RBCs, and impair hemoglobin’s ability to carry and transfer nitrosative equivalents (Kobayashi et al., 2022). These abnormalities weaken the RBC/endothelium communication and diminish nitrosative support for EDH. Whether RBC-derived nitrosative signaling directly sustains the S-nitrosation of endothelial targets, including GC1, remains unknown and has not been directly tested. Nevertheless, this framework raises the possibility that RBCs influence redox-sensitive signaling pathways governing endothelial excitability and electrical behavior. Thus, RBCs should be viewed as central modulators of endothelial redox biology and electrical behavior.

## Conclusion

Taken together, the evidence reviewed here helps resolve a long-standing ambiguity in vascular biology. Nitric oxide does not operate as a single, uniform vasodilator across the arterial tree. Instead, its function is shaped by vessel size, cellular organization, and the surrounding redox environment. In large conduit arteries, NO primarily behaves as a freely diffusible gas that activates GC1 in vascular smooth muscle, leading to cGMP production and PKG activation. This pathway governs smooth muscle relaxation, vascular compliance, and the handling of pulsatile blood flow.

In contrast, vasomotor regulation in resistance arteries, the principal determinants of peripheral resistance and blood pressure, relies predominantly on EDH. This mechanism is fundamentally electrical in nature and depends on localized Ca^2+^ signaling, myoendothelial communication, and activation of KCa channels. In this context, NO assumes a markedly different role. Rather than acting as a direct relaxant, it functions primarily as a redox signal that modulates endothelial excitability through protein S-nitrosation and transnitrosation.

Within the microcirculation, connexins, TRP channels, Ca^2+^-handling proteins, and KCa channels emerge as key redox-sensitive effectors that shape the initiation, amplification, and spread of hyperpolarization. In this setting, GC1 is repositioned from a smooth muscle enzyme to an endothelial redox regulator that coordinates spatially restricted nitrosothiol signaling independently of its canonical cGMP-generating activity. This endothelial GC1-dependent redox program appears critical for preserving Ca^2+^ microdomains and electrical fidelity underlying EDH.

Importantly, EDH does not arise solely from intrinsic endothelial mechanisms but reflects the integration of signals from the vascular microenvironment. PVAT and RBCs provide metabolic, oxygen-dependent, and redox inputs that shape endothelial nitrosative balance and electrical behavior. Disruption of these interactions through metabolic stress, inflammation, or oxidative imbalance selectively impairs EDH, even when NO-cGMP signaling in conduit arteries remains preserved.

A critical and emerging challenge is to identify the specific endothelial targets of GC1 that regulate Ca^2+^ signaling microdomains, electrical coupling, and hyperpolarization. Defining these targets will be essential for developing therapeutic strategies aimed at restoring endothelial and microvascular function, offering a more precise alternative to approaches that simply augment NO availability.
